# The Inflammatory Conspiracy in Multiple Sclerosis: A Crossroads of Clues and Insights through Mast Cells, Platelets, Inflammation, Gut Microbiota, Mood Disorders and Stem Cells

**DOI:** 10.3390/ijms23063253

**Published:** 2022-03-17

**Authors:** Massimo Cocchi, Elisabetta Mondo, Marcello Romeo, Giovanna Traina

**Affiliations:** 1 Department of Veterinary Medical Sciences, University of Bologna, Ozzano dell’Emilia, 40064 Bologna, Italy; massimo.cocchi@unibo.it (M.C.); elisabetta.mondo2@unibo.it (E.M.); 2 Department of Biology and Biotechnology, University of Pavia, 27100 Pavia, Italy; drmarcelloromeo@gmail.com; 3 Department of Pharmaceutical Sciences, University of Perugia, 06126 Perugia, Italy

**Keywords:** multiple sclerosis, mast cells, platelets, inflammation, gut microbiota, mood disorders, stem cells

## Abstract

Multiple Sclerosis is a chronic neurological disease characterized by demyelination and axonal loss. This pathology, still largely of unknown etiology, carries within it a complex series of etiopathogenetic components of which it is difficult to trace the origin. An inflammatory state is likely to be the basis of the pathology. Crucial elements of the inflammatory process are the interactions between platelets and mast cells as well as the bacterial component of the intestinal microbiota. In addition, the involvement of mast cells in autoimmune demyelinating diseases has been shown. The present work tries to hang up on that Ariadne’s thread which, in the molecular complexity of the interactions between mast cells, platelets, microbiota and inflammation, characterizes Multiple Sclerosis and attempts to bring the pathology back to the causal determinism of psychopathological phenomenology. Therefore, we consider the possibility that the original error of Multiple Sclerosis can be investigated in the genetic origin of the depressive pathology.

## 1. Introduction

From the National Multiple Sclerosis Society (New York): The cause of Multiple Sclerosis is still unknown. Scientists believe that a combination of environmental and genetic factors contribute to the risk of developing Multiple Sclerosis.

*“Multiple sclerosis (MS) involves an immune-mediated process in which an abnormal response of the body’s immune system is directed against the central nervous system (CNS). The CNS is made up of the brain, spinal cord and optic nerves”.* This work tries to hang up on that Ariadne’s thread which, in the molecular complexity of the interactions between mast cells, platelets, microbiota and inflammation, characterizes Multiple Sclerosis and attempts to bring the pathology back to the causal determinism of psychopathological phenomenology. Therefore, we consider the possibility that the original error of Multiple Sclerosis can be investigated in the genetic origin of the depressive pathology.

MS is a chronic neurological disease characterized by demyelination and axonal loss. This pathology, still largely of unknown etiology, carries within it a complex series of etiopathogenetic components of which it is difficult to trace the origin. MS is known to be a very complex autoimmune disease involving multiple stages, a wide variety of cell types and a plethora of mediators.

What is certain is that MS is fundamentally characterized by a powerful inflammatory state [1]. Studies have reported that people with inflammatory disorders are prone to depression. There is evidence that inflammatory cytokines can alter neuronal activity as well as mood, although it is still unclear how inflammation contributes to the depressive condition [2]. On the other hand, depression is a common psychiatric feature in MS patients and is accompanied by a deep sense of helplessness and reduced social participation [3]. Epidemiological data report that suicide cases are statistically higher in MS patients [4].

In addition, the role played by the intestinal microbiota (i.e., the community of microorganisms that live in mutualistic symbiosis with its host) on many functions is now widely recognized. In depressed patients, gut microbiota composition has been detected. Furthermore, studies on animal models have shown that the microbiota modulates anxiety and the onset of neurological diseases associated with circuit dysfunctions [5]. However, the degree of changes in the function and composition of the gastrointestinal microbiota that lead to depression and the causal connection of both bacterial commensals and depression have yet to be fully understood to define the role of the gut microbiome in depression.

Starting from this consideration, it can be considered of interest to retrace the salient notes characterizing the “silent” inflammatory process which sees the basic elements of the inflammatory process as the first actors, that is, the interactions between platelets and mast cells and the functional bacterial component of the intestinal microbiota.

Other elements of consideration concern the relationship between “depression” and multiple sclerosis in the evidence, for depression, of the influence of a specific coexistence with the inflammatory process.

In this context, it is also important to emphasize that a convergence of mast cell, platelet, microglia, gut microbiota condition, and underlying inflammatory state has been described in other neurodegenerative diseases [6,7].

## 2. Mast Cells and Multiple Sclerosis

Mast cells (MCs) are innate immunity cells located in mucous membranes and connective tissue, strategically placed at the interface with our external environment such as the skin, lungs and intestines, where they act as gatekeepers for the attack of pathogens [8,9].

Their activation causes the release in a very short time of mediators classified as dependent or independent of degranulation. These mediators contribute to inflammation and changes at the site of infection. There are other responses as well, a storm of chemokines and cytokines that promote the local recruitment of effector cells. IFN is also acting in an autocrine way to further promote the production of MC brokers. The products released by the MCs also act by improving the hypertrophy of the lymph nodes. The involvement of local dendritic cells promotes the development of a subsequent acquired immune response [10]. MCs play a leading role in various pathophysiological conditions, where a condition of chronic silent inflammation is present. IL-1β, IL-6 and IL-8 are representative of silent chronic inflammation, and MCs are producers and effectors of these cytokines. MCs are considered to be fundamental players in immunopathogenesis and so on as potential therapeutic targets in different neural pathologies (for reviews, see [11,12].

MCs are involved in inflammatory responses as well as in psychological stress [11]. MCs play a crucial role in host-microbiota communication, as they can help influence microbiota status and host conditions by modifying their activation [12]. Studies have reported that MCs establish functional signaling pathways with the nervous system and nerves in the gut. The activation of MCs induces sensitization of the nerves, and these in turn can activate or inhibit the release of mediators from MCs. This dialogue plays an important role in the generation of symptoms or even in the pathogenesis of inflammatory disorders [13].

Relationships established between gut microbiota composition, cytokine storms, and MC activation suggest that the gut microbiome is extremely involved in the severity of diseases involving the inflammatory process [9]. MCs can contribute significantly to intestinal homeostasis, and their activation is linked to modifications, motor abnormalities and dysfunctions of the intestinal epithelial barrier [8].

### 2.1. Mast Cells: From Blood to Brain

The demonstration of the presence of MCs in the brains of many animal species, including humans, dates back to a long time ago [14,15,16,17], not to mention Neumann who, in 1890 [17], identified them in infarcted brains and on the edge of multiple sclerosis plaques. The involvement of MCs in autoimmune demyelinating diseases has also been shown in the experimental animal, [18,19,20] due to the ability of neutral proteases to degrade myelin. Cytokines are involved in myelin destruction and remyelination and repair, and a very close relationship between inflammation and exacerbation of MS has been reported [21].

MCs can degranulate in response to a basic myelin protein inducing central and peripheral demyelination [22,23,24]. Levels of tryptase and protease are increased in the cerebrospinal fluid of MS patients and histamine is elevated in their blood [9,24]. In the mammalian brain, MCs are found in the leptomeninges [14,15,25] and are concentrated in the brain parenchyma along the blood vessels of the nuclei of the dorsal thalamus [26] on the brain side of the blood-brain barrier [27,28,29,30]. Studies support the substantial contribution of MCs to BBB disruption, the recruitment of inflammatory cells to the CNS, and local degeneration [24].

MCs communicate with neurons, astrocytes, microglia, the extracellular matrix, and blood vessels [29]. In the absence of stress, disease and traumatic events, MCs are far outnumbered by neurons, microglia, and other brain-resident cells. There is evidence that MCs can penetrate the blood-brain-barrier and break its integrity and that mature ones can transfer from the blood to the brain [30]. The breakdown of the blood-brain-barrier and the degradation of the basal lamina can be traced back to components of MCs such as heparin, histamine, serotonin, nitric oxide, VIP, CGRP (calcitonin gene-related peptide), the endothelial growth factor, the cytokines, and the TNF-α factor which, in turn, induces the expression of the cell adhesion molecule and allows leukocytes to enter the affected tissues [9,31,32,33].

The production of TNF-α by MCs seems to precede its finding in other cells [32].

The MCs tryptase activates microglia receptors 2 (PAR 2), facilitating the release of pro-inflammatory mediators such as TNF-α, IL-6 and reactive oxygen species which, consequently, upregulate the PAR2 expression of receptors on MCs [9,34]. MCs and astrocytes are found in the perivascular areas and in the thalamus. The reciprocity of activation, in vitro, of astrocytes and MCs causes the release of histamine, leukotrienes and cytokines, the latter of which, in turn, can induce the degranulation of MCs [35,36,37].

In this context, levels of tryptase are elevated in the cerebrospinal fluid in MS disease [38].

It seems that the role of MCs in the induction and involvement of the inflammatory process in the brain is not entirely certain or clear.

It is the opinion of the authors, however, that the occurrence of an inflammatory involvement of MCs in the brain is an element to be taken into consideration. In particular, the increase in arachidonic acid (AA) in the brain during depression could be justified by MCs, as well as their pro-inflammatory effect, a condition that accompanies the depressive phenomenon [29,39,40,41]. MCs and nerves communicate with each other [42,43]. Neuronal mechanisms are involved in the activation of MCs that mediate information between peripheral nerves and local inflammatory events. There is an anatomical association between MCs and nerves in many tissues [44,45,46]. Tissue MCs can also be activated under normal conditions, and this suggests a constant flow of information to the nervous system [47]. Even in vivo, there is a functional and two-way communication path.

The activity of MCs in health and disease conditions configures elements of great complexity. The secretory activities of MCs can also occur without degranulation and the molecular content can be released in discriminating ways [48]. MCs can also be substantially involved in inflammatory processes in which degranulation phenomena are not commonly observed. The existence of the activation of the granules contained in MCs or of fragmentary degranulation, in association with differential secretions, has been demonstrated ultra-structurally [49]. IL-1 stimulates IL-6 secretion in the absence of the release of tryptase, a protease associated with granules, and the secretion of IL-6 from MCs, an event that seems to be distinct from the phenomenon of degranulation, contributes to the development of inflammation [50].

Serotonin can be released separately from histamine, and the production of prostaglandins and leukotrienes from AA has also been demonstrated [51]. Studies reported a correlation between the number and/or distribution of MCs and MS or the animal model of experimental autoimmune encephalomyelitis (EAE) pathology. Sites of inflammatory demyelination are also sites of MC accumulation in the brain and spinal cord, and the percentage of degranulated MCs in the CNS correlates with the clinical onset of disease symptoms in acute [52,53,54].

### 2.2. MCs, Brain Inflammation, Psychiatric Disorders

MCs play a significant role in brain pathophysiology by interacting with the glia, endothelial cells and neurons [55]. Stress and CRF (Corticotropin-releasing factor) can activate brain MCs [56]. On the other hand, MCs can release CRH that can affect the blood-brain barrier permeability and activate glial cells to release additional inflammatory mediators in a kind of positive feedback, thus contributing to chronic neuroinflammation in the brain [8,26]. The role of pro-inflammatory cytokines in the brain has been connected to the pathogenesis of psychiatric diseases, major depression, bipolar disorder, and autism spectrum disorders [9,29,57,58,59,60,61] in particular. In this context, evidence suggests that the stabilization of MCs could be a promising treatment for MS patients [24].

In particular, the protective role of MC stabilizer drugs with normalization of the blood-brain-barrier with consequent reduction of MS and EAE has been reported [24,62], although there has been evidence describing an ambiguous scenario regarding the involvement of MCs in the pathogenesis of EAE [63].

The mediation of MCs on anxious behavior demonstrates their action at a central level, while the pharmacological blocking of MCs’ activity at the peripheral level does not change the anxious behavior [64].

In conditions of food allergy, asthma and irritable bowel, pathologies significantly mediated by MCs, there is a significant relationship with anxiety disorders that should not be confused with recurrent episodes of anxiety [65,66,67].

The changes in behavior induced by MCs are attributable to multiple neurochemical interactions, including, for example, the regulation of the sleep-wake cycle mediated by histamine, as occurs in sexual behavior and anxiety [68,69,70,71].

Serotonin, as a neurotransmitter, affects aggression, appetite and mood, and as a trophic factor it affects neurogenesis, emotionality and memory [72,73]. The serotonin depletion of MCs can affect anxious behavior. Cytokines originating from MCs such as TNF-α, IL-1 and IL-6 can act on the hypothalamic–pituitary–adrenal (HPA) axis and stress behavior [74]. In addition, substances derived from MCs, including TNF-α and prostaglandins D2, can perform a neuro-modulatory function by contributing to the regulation of sleep, pain and body temperature [75,76,77,78]. Brain inflammation is therefore involved in the pathogenesis of neuro-psychiatric pathologies, where neurogenic factors can stimulate cerebral MCs to release inflammatory and neurotoxic mediators that alter the permeability of the blood-brain-barrier, stimulate microglia and cause focal inflammation.

In other neurodegenerative diseases, such as amyotrophic lateral sclerosis (ALS), MCs microglia and platelets are involved. In particular, in ALS, a motor neuron disease, which is the third most common neurodegenerative disease after Alzheimer’s disease and Parkinson’s disease, microglia activation has been shown both in transgenic animal models and in the *post-mortem* human brain and also in in vivo imaging in ALS patients [79,80].

On the other hand, treatment with an MC inhibitor reduced the number of MCs and the progression of motor symptoms. In addition, elevated IL-6 and IL-8 levels were found in the peripheral blood of ALS patients [79,80,81].

However, MCs expressing IL-17 were present in the spinal cords of ALS patients, and the MC chemoattractant, IL-15, is elevated in the serum and cerebrospinal fluid of ALS patients [7].

## 3. Platelets

Platelets also play an important role in a variety of regulatory and degenerative processes [82]. Studies report that platelets participate in inflammation by producing a variety of pro-inflammatory molecules [83].

Circulating IL-1β, IL-6 and IL-8 are not regulated in chronic systemic and silent inflammation and also have receptors on platelets [84]. Platelets express a variety of immunologically relevant ligands and receptors, adhering with endothelial cells, monocytes and neutrophils and toll-like receptor- (TLR) mediated responses [85].

Platelets are known for their role in haemostasis, and hypercoagulability is an important sign of inflammation. IL-1β, IL-6, and IL-8 are critically involved in the formation of abnormal clots, erythrocyte pathology and hyperplastic activation [29]. The most important changes were observed when all three cytokines caused platelet hyper-activation and spread with vessel damage and thrombogenic effects. Interestingly, a metabolite derived from the gut microbiota has recently been identified. It is called phenyl-acetyl-glutamine and can improve the phenotypes related to platelet activation, thus favouring platelet hyper-activation. This metabolite could therefore increase thrombotic capacity and, ultimately, increase the risk of cardiovascular complications [86].

Cytokine storms destroy the progenitor cells and lead to reduced platelet production. The immune system destroys platelets, and platelet aggregation occurs in organs, resulting in the consumption of micro-thrombi and platelets [86]. In addition, the production of cytokines is induced by a dysbiotic microbiota, the activating effect that inflammatory stimuli exert on platelets, MCs and astrocytes. This allows for the release, in turn, of other pro-inflammatory substances and certainly results in a multiplication of the harmful effect to the micro-thrombi and, considering the omnipresent position of MCs around the nerves, any neurological and brain damage, up to psychopathological conditions, anxiety and depressive syndromes.

Platelets have a relationship with MS and are involved in the pathophysiology of the disease and interact with endothelial cells, where there are infiltrates of lymphocytes and macrophages. The complexity of these interactions results in damage to the myelin and axons, albeit with mechanisms of still uncertain interpretation [87].

It has also been shown that platelet aggregates are in proximity to perivascular MCs and that the activation of MC is a consequence of platelet activation, which triggers local and systemic responses through the activation of perivascular MC [88].

Platelets, adhering to the endothelium, can influence the inflammatory process at the neuromuscular level through mechanisms that involve the recruitment/activation of leukocyte cells and microglia, as well as the release of chemokines, cytokines, etc. [89,90,91].

Furthermore, platelets enter a relationship with aspects of psychopathology. They also share various similarities with the monoaminergic system [92].

Platelet abnormalities of serotonin absorption, storage and metabolism have been found in the serotonergic and noradrenergic systems of subjects with depressive psychopathology, as well as increased reactivity [93].

Studies have reported an increase of platelet activation in subjects with depression [94].

Other authors have identified a key aspect between platelet fatty acids and psychopathology using a complex mathematical function (Self Organizing Map, SOM) which has allowed them to classify some of the main psychopathologies, such as major depression and bipolar disorder. The results showed that the mobility of the platelet membrane is a critical element in the serotonin uptake in the two different psychopathologies [39,60,95], confirming the similarity between neurons and platelets [96,97].

Furthermore, motor neuron degeneration may be caused by a plethora of pathways. Platelet-activating factor (PAF) is a crucial mediator of inflammatory response that is involved in several leukocyte functions, platelet aggregation and degranulation, and inflammation. In particular, Briones et al. (2018) in a pilot study, hypothesize a possible role for PAF receptor inhibitors as a novel therapy for ALS [98].

## 4. Multiple Sclerosis and Intestinal Microbiota

A further decisive condition in the conditioning of the inflammatory response of the organism certainly sees the involvement of the intestinal microbiota which, at the level of the nervous system, can lead or be accompanied by structural and functional modifications, never forgetting what William P. Hanage reported and which is valid for every scientific novelty that signify an era [99]: “The history of science is full of examples of exciting new fields that have promised a flow of gold for drugs and health insights but have required skepticism and years of effort for even a partial offer of results.”

Several lines of evidence support the thesis that, in central neurodegenerative diseases, imbalances of the brain-gut microbiota axis could lead to the occurrence of neuroinflammatory intestinal conditions and gastrointestinal dysfunction [100].

Others, in line with Braak’s hypothesis on the pathogenesis of Parkinson’s disease, state that central neurodegenerative diseases may have their onset in the enteric nervous system (ENS) and then progressively spread to the central nervous system (CNS) through the nerve pathways that connect the gut to the brain (i.e., the vagus nerve) [101].

The intestinal microbiota, in recent years, has also been extensively studied in relation to MS. In particular, a study [102] has highlighted the presence of bacteria in the brains of subjects with MS. Within the demyelinated areas in subjects with MS and MS models in non-human primates, the peptidoglycan constituting the bacterial cell wall (PGN) has been detected in phagocytes [103,104]. Additionally, a component of PGN, the muramyl dipeptide, known to affect demyelination [105], has been recognized as an inducer of NOD2 and NLRP3 inflammasome in human microglia [106].

The sum of the research on the intestinal microbiota in subjects with MS, compared to healthy controls, revealed conditions of intestinal dysbiosis with significant alterations in the relationships between bacterial species [107]. Overall, MS microbiota studies suggest that there is a depletion of bacteria with the ability to induce cells with immune-regulatory abilities and enrichment of bacteria with the ability to induce pro-inflammatory responses.

In particular, it has recently been shown that subjects with MS have a significantly different intestinal microbiota profile compared to healthy subjects, with a higher representation of *Pseudomonas*, *Methanobrevibacter* (Euryarchaeota phylum) and *Akkermansia* (Verrucomicrobia phylum), and a lower representation of *Parabacteroides*, *Adlerkreutzia Butyricimonas* and *Prevotella*, with possible pro-systemic inflammatory correlated with the gene expression of interferon signaling, dendritic cell maturation, and NF-kB signaling pathways in circulating monocytes and T cells [108].

In conclusion, patients with MS usually have gut dysbiosis and often reduced numbers of *Faecalibacterium*, Bacteroidaceae, and *Prevotella*.

There are also data regarding major depressive disorder, according to which the intestinal microbiota of patients clustered significantly differently than that of healthy controls, with less representation of *Bacteroidetes* and the expansion of *Actinobacteria* [109].

Further research will need to be conducted to determine the role of the gut microbiota and their metabolites in MS susceptibility and protection.

## 5. Ariadne’s Thread of Multiple Sclerosis

We have seen how MS represents a crossroads of mechanisms that induce inflammation, and how these mechanisms interact with each other and are inextricably linked to the disease.

To complete this series of clues, an aspect that has been little debated until now is highlighted, namely the possibility of tracing the error characterizing Multiple Sclerosis to the psychopathological phenomenon of depression as well as the possibility of finding new indications of connection through the study of stem error, which induces the depressive state.

To do this it is necessary to refer to some concepts that can clarify this passage, recalling the study performed on a pathology similar to MS, that of scleroderma. In 1986, some authors [110] identified the similarities and coincidences between the two pathologies.

These authors noted that:

“… Immunological abnormalities in cerebrospinal fluid are remarkably similar in scleroderma involving the central nervous system and in multiple sclerosis. In both conditions, a higher concentration of IgG in the cerebrospinal fluid and of the immune complex in the gamma region was observed on agarose gel electrophoresis. However, immunoglobulin deposition in brain tissue was not consistently found [111]…”

Not surprisingly, multiple sclerosis and systemic sclerosis, two disorders of suspected autoimmune origin, sometimes coincide. This association could be more common than what has been recognized to date “… Although of unknown significance, auto-antibodies against myelin basic proteins, glycolipids and oligodendroglia have been described in Multiple Sclerosis … [112]”.

Again, in 1989, the authors [113] describing the study of a case report that:

“… Careful evaluation of neurological signs in patients with systemic sclerosis may reveal a more frequent co-occurrence of these two diseases, although the association of these two diseases has rarely been reported in the literature …”

We certainly cannot say that these works represent the apotheosis of the relationship between multiple sclerosis and scleroderma, however, they represent that further clue that allows us to reason on the links between the two diseases and depression.

**Figure 1:**
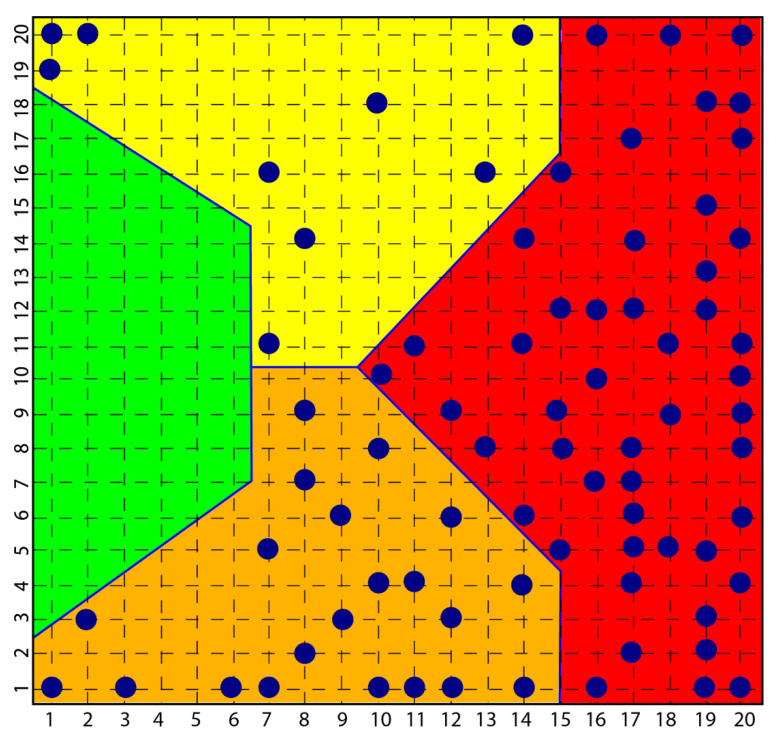
Figure shows how all subjects with psychopathology are distributed among the yellow, orange and red areas and none in the green area that classified normal subjects.

In the course of the aforementioned research, which led to the diagnostic identification of various aspects of psychopathology, in particular, a consistent group of subjects with scleroderma was investigated and the result was that all these subjects were classified in the psychopathological area of the map obtained with a self-organized network [114], Kohonen’s Self Organizing Map SOM [115], highlighting how clear the link between the disease and the depressive phenomenon was and how clear the origin of scleroderma from depression and not vice versa was equally clear (Figure 1).

**Figure 2:**
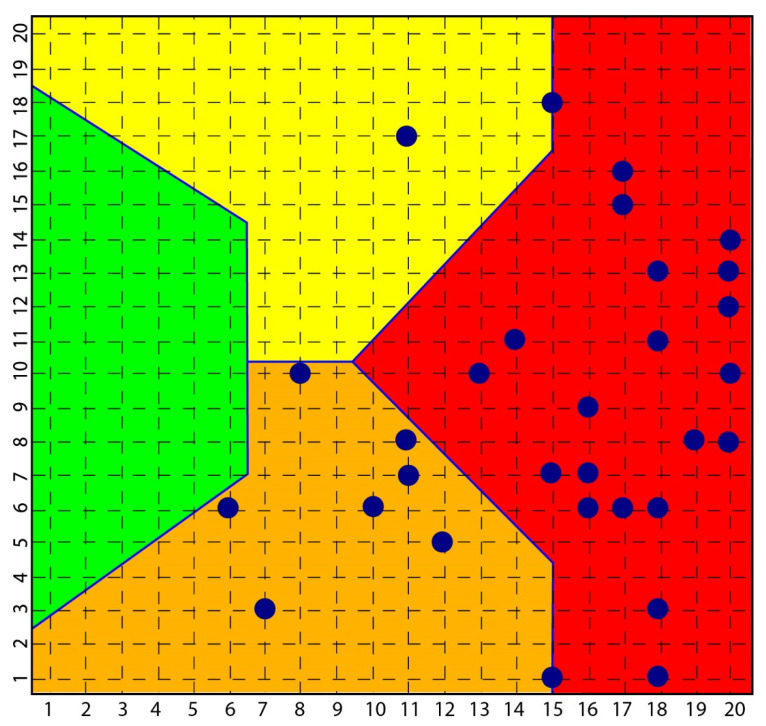
Distribution of scleroderma subjects in SOM. The figure shows how all subjects [35] with scleroderma are distributed among the yellow, orange and red area and none in the green area that classified normal subjects.

According to the strong classifying power of the SOM, all subjects with scleroderma were classified in the context of mood pathology, while none of the subjects investigated with depression and bipolarity had symptoms or signs of scleroderma (Figure 2).

Concerning MS and, as previously described, tracing the triggering causes is increasingly intriguing and obscure, with the awareness that the recognition of the link between psychiatric symptoms and MS was known thanks to Charcot, who gave the first detailed clinical pathological description of “disseminated sclerosis” in his lectures at the Salpêtrière hospital in the nineteenth century [116]. Among the psychiatric symptoms noted by Charcot were pathological laughter, crying, euphoria, mania, hallucinations and depression. Indeed, Charcot’s patient Mademoiselle V has been described as suffering from a form of lypemania (or severe depression), along with hallucinations and paranoia [117]. In the 1950s, real research began to establish the frequency of the depressive phenomenon in MS [118]. In addition to the neurological phenomena that characterize MS, the depressive phenomenon seems to account for 50%. Patten et al. reported a 12-month prevalence rate of 25.7% for major depression in people with MS aged 18–45 years [119]. Of further concern is the finding that suicidal ideation is relatively common among people with MS and that depression in people with MS is often not detected and treated [120,121].

## 6. Conclusions

An extensive examination of the scientific literature confirms that inflammation seems to represent the strongest and most common element of the aforementioned diseases and neurodegeneration in general.

The consideration that we make in relation to the relationship between MS and depressive psychopathology is and remains a hypothesis, as is expressed in the text, in the light of the research that has confirmed the strong incidence of the depressive phenomenon in MS and other degenerative pathologies.

Also in the light of the strong molecular evidence that characterizes the psychopathological aspect of scleroderma, the possibility of a common and original genetic aspect that links depressive psychopathology to neurodegeneration in general and to MS in particular seems to be plausible. It seems, therefore, reasonable, in light of the similar psychopathological aspects in MS and Scleroderma, to deduce that depressive psychopathology could be the common umbrella of multiple sclerosis as evidenced for scleroderma.

These considerations led the authors to formulate the innovative hypothesis of deepening research on the possibility of a common genetic error concerning depressive psychopathology and MS, specifically, the common Ariadne’s thread.

**Figure 3:**
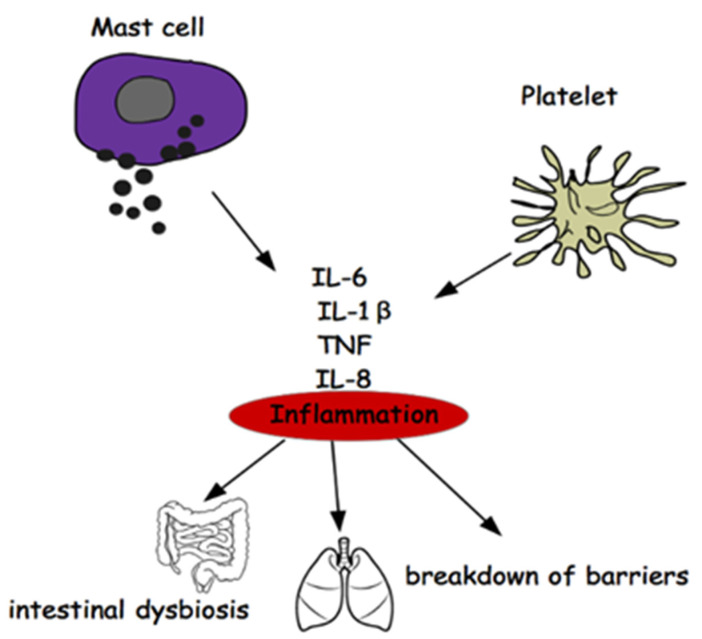
Relationship between Mast Cells, Platelets, Inflammation, Gut Microbiota.

It would therefore be a question of not investigating the disease (MS) as it manifests itself and beyond the inflammation common to all neurodegenerations (Figure 3), but rather on the mechanisms of the differentiation of stem cells that could carry the error that induces the phenomenon of mood disorders, deepening the research on the genetic origin of depression in the light of recent acquisitions obtained on genetic variants associated with depression [122].

## Data Availability

Not applicable.
